# A Computational Systems Pharmacology Approach to Investigate Molecular Mechanisms of Herbal Formula Tian-Ma-Gou-Teng-Yin for Treatment of Alzheimer’s Disease

**DOI:** 10.3389/fphar.2018.00668

**Published:** 2018-06-26

**Authors:** Tianduanyi Wang, Zengrui Wu, Lixia Sun, Weihua Li, Guixia Liu, Yun Tang

**Affiliations:** Shanghai Key Laboratory of New Drug Design, School of Pharmacy, East China University of Science and Technology, Shanghai, China

**Keywords:** traditional Chinese medicine, compound–protein interactions, network-based inference, computational systems pharmacology, Alzheimer’s disease

## Abstract

Traditional Chinese medicine (TCM) is typically prescribed as formula to treat certain symptoms. A TCM formula contains hundreds of chemical components, which makes it complicated to elucidate the molecular mechanisms of TCM. Here, we proposed a computational systems pharmacology approach consisting of network link prediction, statistical analysis, and bioinformatics tools to investigate the molecular mechanisms of TCM formulae. Taking formula Tian-Ma-Gou-Teng-Yin as an example, which shows pharmacological effects on Alzheimer’s disease (AD) and its mechanism is unclear, we first identified 494 formula components together with corresponding 178 known targets, and then predicted 364 potential targets for these components with our balanced substructure-drug–target network-based inference method. With Fisher’s exact test and statistical analysis we identified 12 compounds to be most significantly related to AD. The target genes of these compounds were further enriched onto pathways involved in AD, such as neuroactive ligand–receptor interaction, serotonergic synapse, inflammatory mediator regulation of transient receptor potential channel and calcium signaling pathway. By regulating key target genes, such as ACHE, HTR2A, NOS2, and TRPA1, the formula could have neuroprotective and anti-neuroinflammatory effects against the progression of AD. Our approach provided a holistic perspective to study the relevance between TCM formulae and diseases, and implied possible pharmacological effects of TCM components.

## Introduction

With more than 5,000-year history, the traditional Chinese medicine (TCM) still plays key roles in the treatment of many diseases and disorders worldwide. However, TCM is usually prescribed as formulae, typically consisting of many herbs in different quantity, in which the composition theory “Monarch, Minister, Assistant and Guide” is observed (Jiang, 2005; Xiong et al., 2013; Zhang A. et al., 2013). Thus a TCM formula contains hundreds of chemical components, which makes it complicated and difficult to elucidate the molecular mechanisms of treatment.

In recent years, as the development of systems biology, network pharmacology has emerged as a new subject for us to understand the complex biological systems from an integrated multi-component network view (Hopkins, 2007, 2008). Network pharmacology is especially advantageous in analyzing ‘multi-compound, multi-target, and multi-effect’ scenario to reveal the molecular relationships among compounds and complex diseases from multiple scales (Zhao et al., 2010; Li et al., 2012). Therefore, it is very helpful for illustrating molecular mechanisms of TCM formulae and finding active constituents from herbs (Hao and Xiao, 2014). There are many studies published to date, such as *Radix Curcumae* formula against cardiovascular diseases (Tao et al., 2013), Qing-Luo-Yin against Rheumatoid arthritis (Zhang B. et al., 2013) and Ge-Gen-Qin-Lian decoction against type 2 diabetes (Li J. et al., 2014).

In our previous study, we developed a network pharmacological method, named network-based inference (NBI), to predict potential drug–target interactions (DTIs) between drugs and targets (Cheng et al., 2012). Because NBI method only could predict potential DTIs within a known drug–target network, we then proposed a new method, entitled substructure-drug–target network-based inference (SDTNBI), to predict potential targets for novel compounds without known targets (Wu et al., 2017). SDTNBI utilizes chemical substructures to bridge the gap between known drug–target network and novel compounds. Recently, we further improved SDTNBI by introducing three parameters (α, β, and γ) into it, namely balanced substructure-drug–target network-based inference (bSDTNBI), to identify potential targets for both old drugs and new chemical entities (Wu et al., 2016). With these methods, we developed computational systems pharmacology/toxicology approaches to investigate the molecular mechanisms of therapeutic effects of known drugs or active compounds, and side effects of known drugs or environmental compounds (Cheng et al., 2013a,b; Li H. et al., 2014; Li et al., 2016; Lu et al., 2015; Wang et al., 2017).

**FIGURE 1:**
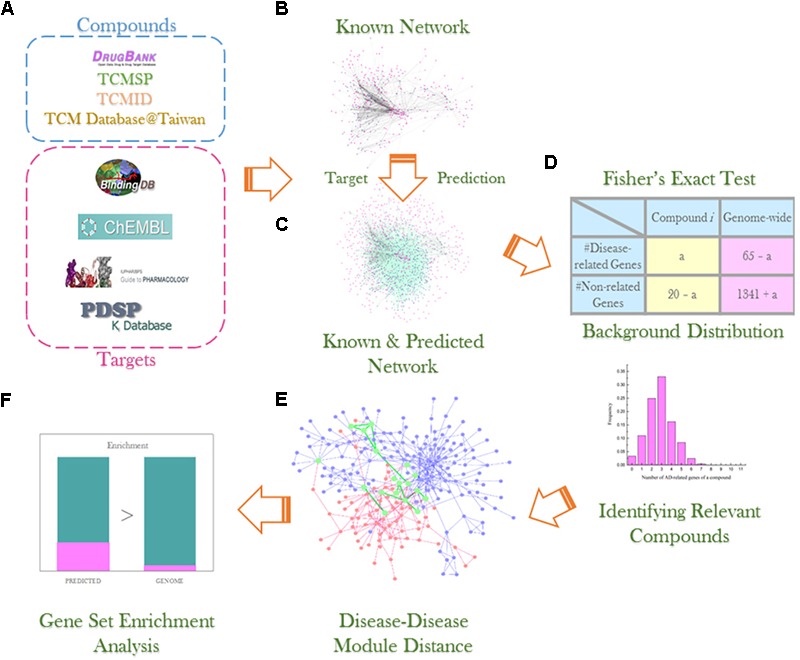
Experiment workflow illustration: **(A)** compound and data collection from multiple databases; **(B)** construction of known compound–target network; **(C)** network link prediction via bSDTNBI method; **(D)** Fisher’s exact test and background distribution to identify compounds significantly related to AD; **(E)** detection of AD and hypertension disease module and analysis of overlapping genes; **(F)** gene set enrichment analysis using DAVID v6.8 to investigate gene sets of interest.

In this study, we proposed a computational systems pharmacology approach (**Figure 1**) combining our bSDTNBI method and statistical analysis to find out the molecular mechanisms of TCM formulae, taking formula Tian-Ma-Gou-Teng-Yin (TMGTY) as an example. TMGTY consists of 11 Chinese herbs, such as *Rhizoma Gastrodiae* (*Tianma*), *Ramulus Uncariae Cum Uncis* (*Gouteng*), *Concha Haliotidis* (*Shijueming*), *Fructus Gardeniae Jasminoidis* (*Zhizi*), and *Radix Scutellariae Baicalensis* (*Huangqin*). TMGTY was prescribed to alleviate hypertension-related symptoms and also showed therapeutic effects against dementia and Alzheimer’s disease (AD) (Liu et al., 2014; May et al., 2016; Zhang et al., 2016; Chen et al., 2017). TMGTY was one of the 10 most commonly used formulae for treating AD in Taiwan, according to a cohort study of one million patients (Lin et al., 2016). TMGTY was also reported to have neuroprotective effects (Chik et al., 2013; Xian et al., 2016) and could enhance the effect of memory acquisition (Ho et al., 2005, 2008). However, its molecular mechanisms remain elusive.

Alzheimer’s disease is a complex neurodegenerative disease that deteriorates memory, cognition, behavior and leads to dementia (Reitz et al., 2011). Several hypotheses were proposed to understand the pathogenesis of AD, including amyloid cascade hypothesis, Tau hypothesis, cholinergic hypothesis and neuroinflammation (Ballard et al., 2011; De Strooper and Karran, 2016; Selkoe and Hardy, 2016). Huge efforts were devoted to the discovery of anti-AD therapies based on these hypotheses, but no curable treatment is available yet. Due to the complex pathology of AD, drugs targeting single protein or pathway may not fully exert expected therapeutic effects. TCM formulae, along with network pharmacology approaches, provide a powerful tool to investigate AD pathology, and they are also promising outsets for anti-AD drug development (Dey et al., 2017; Lai et al., 2017).

For that purpose, we first collected chemical components of TMGTY from related databases, and predicted potential targets for the principal components. Those components were subsequently enriched onto AD-related pathways to find the most disease-relevant component groups. Then we applied gene set enrichment analysis on the groups to examine involved pathways and potential mechanisms in treatment of AD. In order to explore shared mechanisms between diseases, we also mapped disease-related proteins to a protein–protein interaction network to find disease modules. Overlapping sub-modules was presumed as shared mechanisms of which compound targets may affect corresponding diseases. Thus our approach provided a holistic perspective to look into combinations of natural compounds and helped to illustrate their molecular mechanisms.

## Materials and Methods

The whole workflow was illustrated in **Figure 1**.

### Data Collection and Preparation

The formula of TMGTY was ascertained through literature survey. For each herb medicine in the formula, its constituents were mapped from TCM Systems Pharmacology Database (TCMSP) (Ru et al., 2014), TCM Integrated Database (TCMID) (Xue et al., 2013) and TCM Database@Taiwan (Chen, 2011). Then two important pharmacokinetic properties: human intestinal absorption (HIA) and blood brain barrier (BBB) penetration were predicted for every ingredient by our widely used webserver admetSAR^1^. Ingredients with HIA and BBB penetration classified as negatives were considered poorly absorbed by intestine and can hardly penetrate the BBB, and hence excluded from further analysis.

Alzheimer’s disease and hypertension related genes were collected from The Comparative Toxicogenomics Database (CTD) (Davis et al., 2017), Human Genome Epidemiology (HuGE) Navigator (Yu et al., 2008), Kyoto Encyclopedia of Genes and Genomes (KEGG) (Kanehisa and Goto, 2000; Kanehisa et al., 2017), Online Mendelian Inheritance in Man (OMIM) (Amberger et al., 2009), and Pharmacogenetics and Pharmacogenomics Knowledge Base (PharmGKB) (Whirl-Carrillo et al., 2012). In CTD, only genes having a curated association to the disease were retained, i.e., genes marked as ‘marker/mechanism’ and/or ‘therapeutic’ in the ‘direct evidence’ column. In HuGE Navigator, genes with more than 20 publications were selected. In KEGG, ‘KEGG Disease’ was used to find disease genes of AD (KEGG Entry ID: H00056) and hypertension (KEGG Entry ID: H01633). In OMIM, associated genes of AD (Phenotype MIM number: 104300) and hypertension (Phenotype MIM number: 145500) were collected. AD (Accession ID: PA443319) and hypertension (Accession ID: PA444552) related genes in PharmGKB were downloaded. The collected genes were filtered using NCBI Gene database (Brown et al., 2015), and only protein-coding genes were retained.

To build network prediction models, a collection of TCM ingredients was made by combining molecules from above-mentioned TCM databases. Small molecules from DrugBank (Wishart et al., 2017) database were also collected.

For all ingredients, MacroModel 11.1 program (Schrödinger, LLC, New York, NY, United States, 2016) was applied to desalt and neutralize their structures. Then Epik 3.5 program (Schrödinger, LLC, New York, NY, United States, 2016) was used to generate tautomers. Only the most populated neutral tautomers were retained. The processed compounds were further converted to a canonical SMILES string by Open Babel toolkit (version 2.3.1) (O’Boyle et al., 2011). Duplicates were removed according to canonical SMILES string. Compounds without carbon atoms were also removed from the collection.

For each ingredient, the corresponding targets were matched from BindingDB (Gilson et al., 2016), ChEMBL (Gaulton et al., 2017), IUPHAR/BPS Guide to PHARMACOLOGY (Harding et al., 2017) and NIMH Psychoactive Drug Screening Program (PDSP) Ki Database (Roth et al., 2000) under criteria that: (1) target proteins are from *Homo sapiens* and have unique UniProt accession numbers; (2) *K*_i_, *K*_d_, IC_50_ or EC_50_ ≤ 10 μM, or Potency ≤ 10 μM with “Activity Comment” marked as “Active.” The Klekota–Roth (KR) fingerprint was used in this study and generated for every ingredient using PaDEL-Descriptor software (version 2.18) (Yap, 2011).

### Construction of Compound–Target Networks

Three compound–target network models were built by our bSDTNBI method for new target prediction. The first was DrugBank network which contains only small molecules from DrugBank. The second was TCM network consisting of sole TCM ingredients from above collections. The last was a Global network merged by above two networks. The three models were evaluated independently to verify whether a combined model would outperform the others.

Ten-fold cross validation was applied to evaluate the performance of three models. In each fold, roughly 10% of DTIs were split from the network, serving as test set. Resources were redistributed among the remaining 90% of network (i.e., training set) to predict the 10% missing links. This process was repeated for ten times to reduce contingency. For three parameters α, β and γ used to tune the network performance, grid optimization was employed to search for the best set which maximizes the AUC of 10-fold cross validation. Detailed definition and description of these three parameters could be found in our previous publication (Wu et al., 2016).

Several indicators were calculated to assess model performance, such as precision (P), recall (R), precision enhancement (e_P_), and recall enhancement (e_R_). Furthermore, receiver operating characteristic (ROC) curves were plotted by true positive rate (TPR) against false positive rate (FPR). In this study, area under ROC curve (AUC) was calculated and used as an indicator to evaluate model performance since AUC is independent of the number of predicted targets. Basically, the higher the AUC value, the better the model performance. Above indicators were described in details and also widely used in previous studies (Cheng et al., 2012; Lü et al., 2012; Wu et al., 2016).

### Target Prediction for TMGTY Ingredients

For all TMGTY ingredients, bSDTNBI was used to infer new targets. Most of the ingredients were not in the global network model, i.e., do not have known targets. They were represented by molecular fingerprints to link to the global network. The method redistributes known initial resources of drugs between different types of nodes to infer new targets. The resource diffusion number was set to 2 and the number of predicted targets was set to 20. The detailed description and evaluation of the method can be referred to our previous published papers (Wu et al., 2016, 2018).

### Identification of AD-Related Components

Considering only a very small portion of natural products have known targets, the number of a compound’s targets related to AD conforms approximately to hypergeometric distribution and its probability mass function is as following:

(1) $$\text{P}(X=k)=\frac{\left(\begin{array}{c} K\\ k \end{array}\right)\left(\begin{array}{c} N-K\\ n-k \end{array}\right)}{\left(\begin{array}{c} N\\ n \end{array}\right)}$$

Where *N* is the total number of genes, *K* is the total number of AD-related genes, *n* is the number of predicted genes, *k* is the number of AD-related genes in predicted genes and P(*X* = *k*) is the probability of *k* AD-related genes occurring in predicted genes for a compound.

However, the constructed network could not cover all protein-coding genes, so in a certain network, *N* was the number of protein-coding genes it covered, *K* was the number of all AD-related genes in the network and *n* was the number of predicted genes.

Fisher’s exact test method was implemented to assess the significance of enrichment of AD-related genes in 20 predicted and known genes for each compound. Compounds with or without known targets were calculated separately. For compounds without known targets, *n* was set to 20 and *k* was the number of AD-related genes in *n*. As for compounds having *s* known targets, *n* was set to 20+*s*, and *k* was *s* plus the number of AD-related genes in *n*. *P*-value was calculated and adjusted by Benjamini–Hochberg method, and used to rank all compounds. Top-ranked compounds were presumed to be critical components of this formula in treating AD.

In order to take into consideration the properties of chemical components, all compounds collected from the three TCM databases were used to create a background component set. Twenty targets were also predicted for each compound. Then every compound had *k* AD-related genes. The frequency distribution of *k* in the background set was calculated and approximated roughly to the probability distribution of the background set:

(2) $$\text{P}(X=k)=\frac{NP_{k}}{NP}$$

Where NP is the number of all collectable compounds and NP*_k_* is the number of compounds having *k* AD-related genes. Then, if a compound has *k* AD-related genes where P(*X* ≥*k*) < 0.01, the compound is probably enriched onto AD. This was considered as a calibration and corroboration to Fisher’s exact test.

### Disease Module Analysis

Due to the complexity of biological network, common proteins may be shared among different diseases. Thus a drug acts on a single protein may produce effect on multiple diseases. The overlap of AD and hypertension disease modules was thus investigated to find common proteins. The collected AD-related proteins and hypertension related proteins were mapped onto a protein–protein interaction network which consisted of 13,460 proteins and 141,296 physical interactions. A disease module was calculated as the largest connected component (LCC) of the disease-related proteins in the protein–protein interaction network. Then the sizes of LCCs of 100,000 randomized protein sets in the network as large as the disease-related protein set were calculated and the distribution yielded. The statistical significance of the disease module was calculated as a *z*-score:

(3) $$z-score=\frac{S-S^{\bar{rand}}}{\sigma (S^{\text{rand}})}$$

Where *S*, 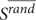, and σ(S^rand^) denote the size of LCC of the disease-related protein set, the average value and standard deviation of the LCC size random distribution, respectively. A *z*-score greater than 1.96 indicates a significance *p*-value < 0.05, which suggests the disease module is larger than random observation. The above used protein–protein interaction network and algorithms were retrieved from the work of Menche et al. (2015).

Then the overlapping proteins of these two modules were extracted and gene set enrichment analysis was conducted to find enriched Gene Ontology biological processes with a cut-off adjusted *p*-value < 0.05.

### Gene Set Enrichment Analysis

Predicted genes and overlapping genes of AD and hypertension modules were enriched onto KEGG Pathway and Gene Ontology (GO) biological process to detect key targets and pathways using the Database for Annotation, Visualization and Integrated Discovery (DAVID) v6.8 (Sherman et al., 2007; Jiao et al., 2012).

## Results

### Formula Ingredients and Known Targets

A total of 731 compounds were collected from the above-mentioned three TCM databases for the formula TMGTY. In order to evaluate the pharmacokinetic properties of this oral administrated formula, HIA and BBB penetrations were predicted using our *in silico* system admetSAR. Only compounds that have both HIA and BBB penetrations predicted as positives were retained. After the screening, 494 herbal compounds were left for further study. 178 different known targets were matched for them from above described databases. Only 68 of 494 compounds had known targets. There were 394 known compound–target interaction pairs. All compounds and known targets information can be found in Supplementary Table S1.

### AD and Hypertension Related Genes

From a series of gene databases, a total of 195 genes were collected and identified as AD-related genes, among which 10 are cytochrome P450 (CYPs). Two hundred and ninety-eight genes were collected as hypertension-related genes, within which nine were CYPs. CYPs are major catalysts contributing to the metabolism of a broad spectrum of endogenous compounds, xenobiotics, and nearly 90% marketing drugs (Guengerich et al., 2016). Due to the substrate promiscuity and wide existence, CYPs were thus excluded from this gene set. Then 185 AD-related genes and 289 hypertension-related genes were used for further experiment. The collected disease associated genes were listed in Supplementary Table S2. Gene set enrichment analyses of these gene groups were also shown in Supplementary Tables S3–S6.

### Compound–Target Networks

**FIGURE 2:**
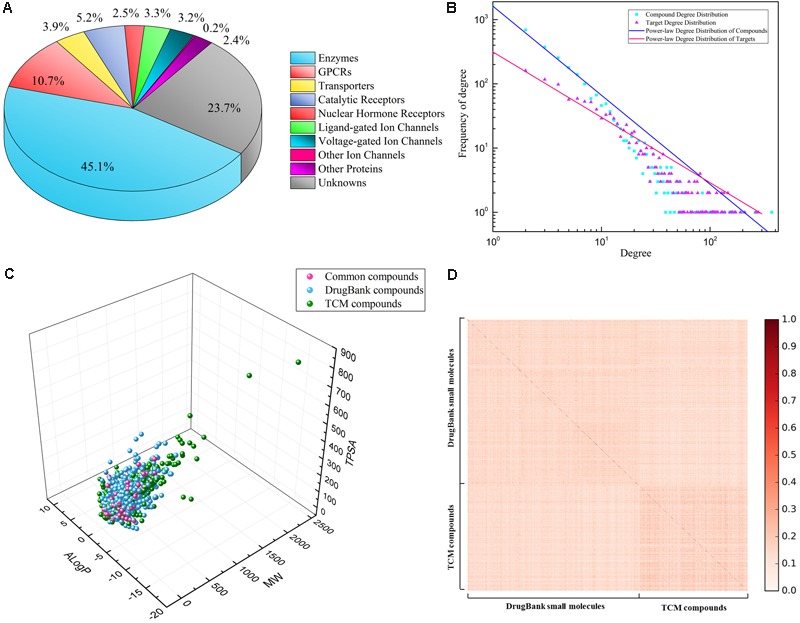
Property analysis of Global network: **(A)** percentage of different kinds of predicted targets, according to IUPHAR classification; **(B)** power-law degree distribution of two kinds of nodes: compounds (blue), targets (red); **(C)** chemical space of all compounds in Global network, depicted by three descriptors: MW, ALogP, and TPSA, consisting of compounds form TCM database (blue), DrugBank database (green), and intersection (red); **(D)** chemical similarity of all compounds on FCFP4 fingerprints.

Three compound–target interaction networks were constructed, namely DrugBank, TCM, and Global networks. Their details were shown in **Table 1**. The TCM network collected 1,495 compounds from TCM databases with 899 known targets, among which 287 compounds were also found in DrugBank network. The DrugBank network contains 2,672 small molecules and 1,326 protein targets. Only one hundred targets in TCM network were different from those in DrugBank network. It was thus combined with DrugBank network to form the Global network in order to introduce more targets and expand known network. In Global network, 45.1% targets were enzymes, 10.7% were GPCRs and about 6.7% were ion channels, while 23.7% fell into unknown category, according to IUPHAR classification (**Figure 2A**). As for compounds in Global network, their chemical space was described using three physicochemical descriptors, i.e., molecular weight, ALogP and topological polar surface area (TPSA), depicted in Figure. 2C. About 95% of all compounds have a molecular weight less than 600, TPSA value less than 200Å^2^ and ALogP value lying in (-3, 3). Only a few TCM compounds have vast values, whose TPSA, for example, could be up to 800 Å^2^. Similarities between all compounds were assessed by calculating Tanimoto coefficient on FCFP4 (**Figure 2D**). Compounds in **Figure 2D** were ordered as two groups: DrugBank small molecules and TCM compounds. Within each group, compounds were randomly distributed. An average Tanimoto coefficient of 0.13 was yielded, indicating a structural diversity among the compounds. The degree distributions of compound nodes and target nodes were calculated, as shown in **Figure 2B**, which succumb to power law distribution. This demonstrated that these networks are scale-free networks.

**Table 1:** Overview of the three compound–target interaction (CTI) networks.

Network	N_C_	N_T_	N_CTI_	Sparsity (%)
DrugBank	2,672	1,326	16,243	0.46
TCM	1,495	899	5,811	0.43
Global	3,880	1,426	19,800	0.36

*N_*C*_, the number of compounds; N_*T*_, the number of targets; N_*CTI*_, the number of CTIs; Sparsity: N_*CTI*_/(N_*C*_ × N_*T*_).*

Our previously developed method bSDTNBI was applied to these three networks. Ten-fold cross validation was then conducted to assess model performance. Parameters α, β, and γ were determined as 0.32, 0.14, and -0.48, respectively. The performance is usually evaluated by several indicators such as AUC, precision and recall. The higher the values of indicators are, the better the performance is. Those indicators were listed in **Table 2** and corresponding ROC curves were plotted in Supplementary Figure S1. All three models performed well. The average recall values of DrugBank, TCM and Global network models were 0.729 ± 0.014, 0.694 ± 0.020, and 0.724 ± 0.012, respectively. A recall value around 70% indicated that, on average, approximately 70% of a drug’s missing links were recovered correctly during 10 rounds of 10-fold cross validation. The value of AUC lies in [0, 1]. AUC value equals to 0.5 means the model gives a random prediction; it equals 1 means an ideal prediction. The AUC values of three above network models were 0.966 ± 0.002, 0.948 ± 0.005, and 0.968 ± 0.002, respectively, all larger than 95%, exhibiting high prediction accuracy. The Global network model outstripped the other two with greater indicator values. The Global network model was thus selected to predict targets for the TCM formula.

**Table 2:** Ten-fold cross validation performance of the three network models.

Network	AUC	Precision	Recall	e_P_	e_R_
DrugBank	0.966 ± 0.002	0.065 ± 0.001	0.729 ± 0.014	42.45 ± 0.81	47.20 ± 0.96
TCM	0.948 ± 0.005	0.049 ± 0.002	0.694 ± 0.020	28.81 ± 0.77	29.94 ± 0.83
Global	0.968 ± 0.002	0.061 ± 0.001	0.724 ± 0.012	46.41 ± 0.75	50.45 ± 0.83

*AUC, area under receiver operating characteristic (ROC) curves; e_*P*_, precision enhancement; e_*R*_, recall enhancement.*

### Target Prediction for Formula TMGTY

Based on the Global network, a total of 9,880 new compound–target interaction pairs were predicted via bSDTNBI method, which introduced 364 new targets for the 494 components of TMGTY. Together with the 178 known targets, a total of 542 targets formed 10,274 interactions with the 494 compounds. Compound–target pairs were hugely complemented from previous 394 interactions between 68 compounds and 178 targets. Among most predicted targets for novel compounds, many were related to AD, such as ACHE, BCHE, BACE1, and MAOA (represented by official gene symbol). A complete list of known and predicted targets for all ingredients can be found in Supplementary Table S7.

### Identification of AD-Related Components

In the Global network, only 1426 proteins were included, among which 65 proteins were AD-related. Thus, in the case of Global network, the probability of a compound having *k* AD-related targets conformed to an approximated hypergeometric distribution which had *K* = 65 and *N* = 1,426.

Then the adjusted *p*-value was proposed as an indicator: the lower is the adjusted *p*-value; the higher is the relevance to AD. A stringent *p*-value (less than 0.01) was used to identify compounds highly related to AD. Based on P(*X* ≥*k*) =0.007 < 0.01, a compound having *k* = 6 or more predicted targets were considered significantly related to AD. For compounds having known targets, Fisher’s exact test was applied separately. Compounds having *p*-values less than 0.01 were also considered enriched onto AD, regardless the number of AD-related targets they have.

Then a real distribution was investigated. A total of 57,741 compounds were collected and processed from TCMSP, TCMID, and TCM Database@Taiwan to serve as a background set. Twenty novel targets were predicted for each compound and the number of AD-related genes was matched using previously collected AD-related genes. The number of AD-related targets of a compound ranged from 0 to 11.

In the background set, the probability of a natural product having *k* AD-related targets was approximated to the real frequency distribution. Only 3.0% natural products of all had 6 or more AD-related targets and 11.4% had 5 or more, in the real distribution (Supplementary Figure S2). Thus, P(*X* ≥ 6) = 0.030 < 0.05 and it further corroborated that a compound having 6 or more AD-related targets was significantly enriched onto AD.

All compounds in the formula were assessed by above two methods. Twelve compounds met the criteria and thus were considered significantly related to AD (**Table 3**). Detailed information on these 12 compounds was listed in Supplementary Table S8.

**Table 3:** The 12 compounds were identified highly related to AD, using a cut-off *p*-value < 0.01.

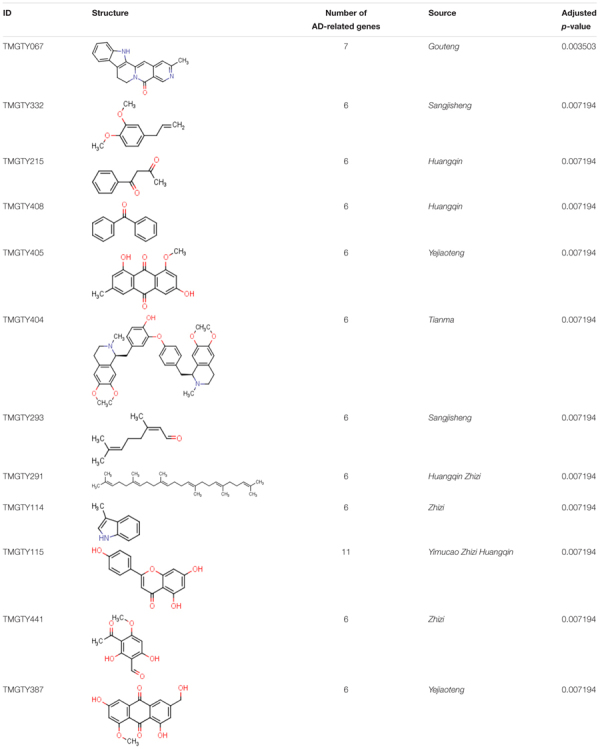

### Pathways Related to TMGTY

**FIGURE 3:**
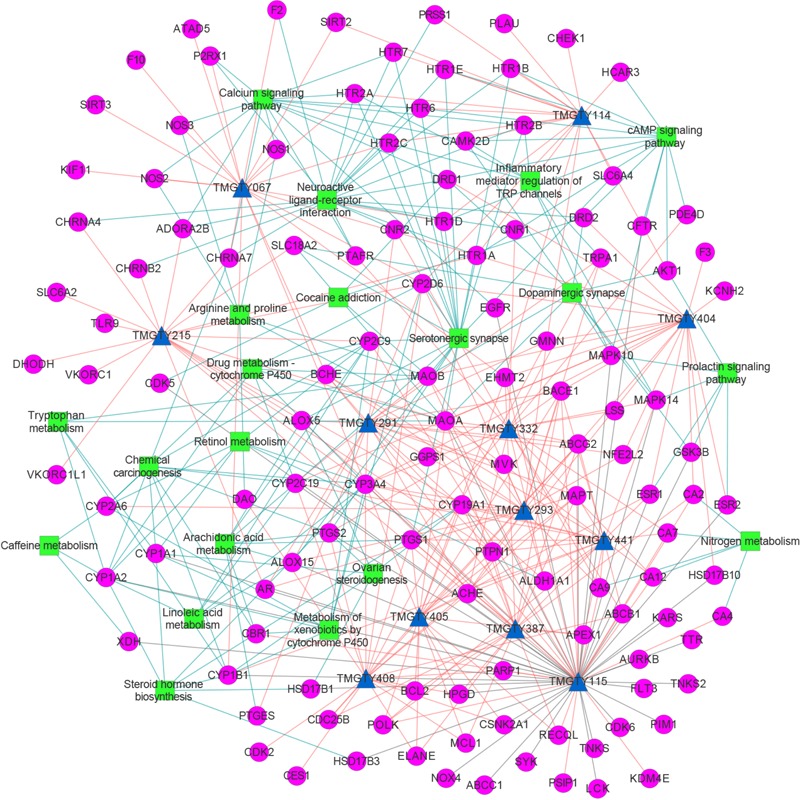
The compound–target–pathway subnetwork of 12 compounds relating to AD. Compound was denoted by blue triangle, target by magenta circle and pathway by green rectangular. Known compound–target interaction was represented as gray line, predicted compound–target interaction as salmon line and target–pathway association as blue line. This figure was plotted using Cytoscape 3.3.0.

All known and predicted genes of these 12 compounds were enriched onto the KEGG pathways. 121 genes were significantly enriched onto 20 pathways with the adjusted *p*-value < 0.05 (Supplementary Figure S3). Many target genes were enriched onto pathways related to neurotransmitters. For example, there were 20 targets enriched onto serotonergic synapse pathway (adjusted *p*-value = 3.5 × 10^-13^) and 21 targets enriched onto neuroactive ligand–receptor interaction pathway (adjusted *p*-value = 3.7 × 10^-7^), while 10 targets were enriched onto dopaminergic synapse pathway (adjusted *p*-value = 2.3 × 10^-3^). There were also 15 targets enriched onto calcium signaling pathway (adjusted *p*-value = 3.7 × 10^-7^), eight onto arachidonic acid metabolism (adjusted *p*-value = 3.7 × 10^-7^) and seven onto inflammatory mediator regulation of TRP channels (adjusted *p*-value = 3.7 × 10^-7^). These pathways may involve in Ca^2+^ regulation and inflammation. Then a compound–target–pathway subnetwork was built according to target prediction and gene enrichment results, as shown in **Figure 3**. In the network, 11 genes had node degrees larger than 5, which indicated that they were potential targets to half or more than half of those 12 representative compounds. These genes were CYP3A4, ACHE, ABCG2, BACE1, CYP2D6, MAPT, MAOA, PTPN1, EHMT2, PTGS1, and CYP19A1.

### Overlap of AD and Hypertension Disease Modules

A total of 172 AD-related proteins and 264 hypertension-related proteins were mapped onto the protein–protein interaction network. AD disease module and hypertension disease module were identified as the LCCs consisting of 86 and 166 proteins, respectively. The *z*-scores of AD and hypertension modules were 14.2 and 11.7, suggesting that the calculated modules were significantly larger than random expectations. Nineteen genes were shared by AD and hypertension, namely ABCB1, AHR, APOE, BCL2, CAT, CRP, ESR1, F2, GPX1, GSK3B, IL1B, LEP, MME, MTOR, NOS2, NOS3, PON1, SOD1, and SOD2. The disease relationship network was shown in Supplementary Figure S4. Then 26 key GO biological processes with *p*-values < 0.05 were identified by conducting gene set enrichment analysis on these 19 genes (Supplementary Figure S5). Biological processes such as response to reactive oxygen species, response to hydrogen peroxide, positive regulation of nitric oxide (NO) biosynthetic process, regulation of blood pressure, removal of superoxide radicals, and NO mediated signal transduction all together indicate that these overlapping genes are related to the progression of hypertension and inflammation.

## Discussion

### The Computational Systems Pharmacology Approach Is Valuable for TCM Study

In this study, a computational systems pharmacology approach consisting of network link prediction, statistical analysis and bioinformatics tools was proposed to study TCM formula TMGTY, which has demonstrated a great advantage in investigation of molecular mechanisms of TCM formulae.

The network link prediction was performed with our bSDTNBI method, which was specifically developed for target prediction of new compounds outside of the compound–target network. Due to the scarcity of TCM ingredient–target interaction information, TCM ingredient–target network was highly incomplete and only covered a small range of targets, i.e., 899 targets, which limited its adaptability and impaired its prediction ability. Since an overlap of 287 compounds was found between collected TCM ingredients and DrugBank small molecules, a combined Global network was then used in order to cover more targets. The combined Global model also outperformed the other two. Comparing to other network prediction models for natural products (Fang et al., 2017a,b) which had approximately 750 drug targets in their global models, our global model covered a much wider range of targets, i.e., 1,426 drug targets, which empowered our model a greater potential to predict more diverse and credible targets for natural products with higher accuracy (using AUC value as an evaluation indicator).

The statistical analysis was conducted by Fisher’s exact test. Comparing to gene set enrichment analysis methods (Huang et al., 2009), hypergeometric distribution was used to enrich AD-related genes on compounds. Natural products were enriched onto AD through AD-related genes using Fisher’s exact test. For each compounds in the formula TMGTY, its known and predicted targets were identified as whether AD related or non-related. From the perspective of our bSDTNBI method, this resource diffusion method can in a way be envisaged as a distance-based similarity method. In the network, the more and the shorter the paths between a compound node and AD-related target nodes, the more resource it would be portioned from AD-related target nodes, i.e., more AD-related targets would be predicted for this compound. This implied certain intrinsic similarities between compounds represented by network topologies. Since the prediction cannot be perfectly accurate, it was reasonable to consider that a compound having more predicted AD-related targets compared to non-related targets was more topologically similar to compounds having known AD-related targets, and thus more relevant to AD. Then Fisher’s exact test was a good statistical tool to assess the relevance.

The cut-off *p*-value for compound selection and further analysis was set to 0.01. Twelve compounds were selected and a total of 121 targets were predicted for them. If the cut-off *p*-value were set to be 0.05, then 108 compounds would meet the criterion and 256 targets predicted. Compounds with a *p*-value between 0.01 and 0.05 were also considered relating to AD. However, 24 out of 121 targets for 12 compounds were AD-related while only 30 out of 256 targets for 108 compounds were AD-related. This suggested that many targets, especially AD-related targets, were shared among compounds with *p*-value less than 0.05. So the compounds with *p*-value less than 0.01 and their corresponding targets were representatives of the molecular mechanisms of formula TMGTY against AD.

The gap between compounds and diseases were bridged by combining above methods. The link between compounds, target genes and diseases was established and analyzed in the context of network science. Complex compound–protein and protein–protein interaction networks were both taken into consideration to facilitate the apprehension of the formula’s mechanisms. Since TCM formulae have complicated constituents and are intrinsically multi-targeted and effective to diverse symptoms, our computational systems pharmacology approach provides a more comprehensive perspective to understand their mechanisms on a systematic level, and is easy to apply to various formulae, thus valuable to TCM study.

### Evaluation of the Systems Network Pharmacology Approach

In order to further validate the performance of our approach, another traditional Chinese herbal formula Kai-Xin-San (KXS) was analyzed using this approach. KXS is a famous formula used for the treatment of neurosis and AD, which consists of four herbs: *Panax ginseng (Renshen), Wolfiporia cocos (Fuling), Polygala tenuifolia (Yuanzhi)*, and *Acorus tatarinowii (Shichangpu)* (Zhou et al., 2012; Wang et al., 2015; Chu et al., 2016).

Three hundred and eighty-eight compounds were collected and 369 new targets predicted, then 28 representative compounds were identified and their targets were enriched onto KEGG pathway and GO biological process (Supplementary Tables S9–S13 and Supplementary Figure S6).

Among enriched pathways, nitrogen metabolism, neuroprotective ligand–receptor interaction, serotonergic synapse, dopaminergic synapse, arachidonic acid metabolism, linoleic acid metabolism, and tryptophan metabolism may be involved in AD pathology. GO biological process enrichment analysis indicated that KXS may involve in oxidation–reduction process, steroid metabolic process, memory, heterocycle metabolic process, lipoxygenase pathway and synaptic transmission, dopaminergic. These enrichment analyses suggested that KXS may exert neuroprotective effect by regulating metabolism networks, reversing oxidative damage in brain, as well as targeting neurotransmitter pathways.

Chu et al. (2016) discovered that KXS could alleviate cognitive deficits in AD model rats and more nerve cells survived than that in the control group. KXS could also regulate metabolism network, such as linoleic acid metabolism and arachidonic acid metabolism, by affecting certain metabolites to show anti-AD effects (Chu et al., 2016). Qiong et al. (2016) found that in rat models KXS could reduce the level of 3-nitro tyrosine (3-NT), and increase the activity of choline acetyltransferase, indicating antioxidant effects of KXS. Zhu et al. (2016a,b) discovered that KXS could induce synaptic protein expression in hippocampus neuron in rats and neuronal differentiation in PC12 cells.

In 28 representative compounds, Apigenin, Paeonol were reported to be important anti-AD compounds (Su et al., 2014). Eudesmin could up-regulate the expression of GABAA and Bcl-2, and it has significant anticonvulsant and sedative effects (Liu et al., 2015). 2′-*O*-Methylisoliquiritigenin was reported to have antioxidant activity and it was also active against human neuroblastoma cells (Batovska and Todorova, 2010). Marmesin was reported to have AChE inhibitory effects (Tumiatti et al., 2008; Cabral et al., 2012). Bergapten was discovered to have anti-inflammatory effects by suppressing the ROS and NO generation (Yang et al., 2018). Myrcene and eugenol was reported to have anti-inflammatory and antioxidant activities (Irie, 2006; Rufino et al., 2015).

The analyses of KXS further validated and proved our approach would be useful in the analyses of TCM formulae and identification of key herbal constituents. Detailed description of KXS can be found in Supplementary Data.

The precision values of three network models were among 0.049–0.065. Different from machine learning methods, bSDTNBI is a network-based method, and its evaluation indicators are from recommender systems (Zhou et al., 2010; Lü et al., 2012). The precision is defined as:

(4) $$\text{P}=\frac{1}{c}\cdot \sum_{i=1}^{\text{c}}\frac{TP_{i}(L)}{L}$$

Where *C* is the number of compounds, *L* is the number of predicted targets and *TP_i_*(*L*) is the number of recovered missing links of compound *i* from test set in *L* targets. Hence, the more targets predicted, the smaller the precision.

The precision value of approximately 0.06 is relatively high (*L* = 20), comparing to previous network-based studies. Fang et al. (2017b) constructed network models with precision values ranged from 0.010 to 0.049. Precision values were among 0.042 to 0.072 in the work of Wu et al. (2016). They tested 56 available compounds predicted to act on estrogen receptor α (ERα), and 27 compounds were identified as active agonists or antagonists (Wu et al., 2016). Wu et al. (2018) also constructed global network models with precision values ranging from 0.045 to 0.055. The comparison also further validated the performance of our approach.

### Potential Mechanisms of TMGTY in Treating AD

In the compound–target–pathway subnetwork, many enriched pathways were related to AD. For example, among target genes enriched onto serotonergic synapse pathway, 5-hydroxytryptamine receptor 2A (5-HT_2A_), 5-hydroxytryptamine receptor 2C (5-HT_2c_), and 5-hydroxytryptamine receptor 6 (5-HT_6_) were reported to be related to AD (Wilkinson et al., 2014). 5-HT_2A_ and 5-HT_2c_ can modulate processing of amyloid protein precursor (APP) (Nitsch et al., 1996). Antagonists of 5-HT_6_ can improve cognitive performance involving stimulation of glutamate, acetylcholine, and catecholamine release in brain (Benhamú et al., 2014). 5-HT_6_ antagonists may also stimulate neurite outgrowth and inhibit mTOR pathway (Claeysen et al., 2015). All 12 compounds were predicted to act on targets in the pathway, mostly 5-HT receptors. Oxidative stress also contributes to neurodegeneration in AD (Barnham et al., 2004). The excessive generation of reactive oxygen species (ROS) leads to free radical-mediated processes harmful to brain cells (Balaban et al., 2005). Monoamine oxidase A (MAO-A) and monoamine oxidase B (MAO-B) are involved in ROS production while catalyzing various amines (Melo et al., 2011). Acetylcholinesterase (AChE) was predicted to potentially interact with 10 compounds, which was also a neurotransmitter receptor. Thus it may also play an important role. Inhibitors of AChE, such as U.S. Food and Drug Administration (FDA) approved drugs donepezil and galantamine can stabilize or slow decline in cognition (Hansen et al., 2008). Efforts are made to develop multi-target drugs to improve therapeutic efficacy. Ladostigil, for example, is a multi-target drug designed to have AChE, butyrylcholinesterase (BChE) and brain selective MAO-A and MAO-B inhibitory activities (Mangialasche et al., 2010). This design strategy suggests an indigenous advantage of TCM formula in treating complex diseases, such as AD in this case: its multi-targeting attribute. Furthermore, compound TMGTY404 (Dauricine), from the main herb *Rhizoma Gastrodiae*, may potentially act on dopamine receptors, which could also help to stabilize neurodegeneration and cognitive decline in AD (Martorana and Koch, 2014). Twenty-one targets enriched onto neuroactive ligand–receptor interaction pathway were mostly neurotransmitter receptors. All above pathways suggested that this formula may exert neuroprotective effect by targeting various neurotransmitter receptors to treat AD.

Moreover, there were other enriched pathways may be involved in AD pathology, such as calcium signaling pathway, inflammatory mediator regulation of TRP channels and arachidonic acid metabolism. Ca^2+^ plays an important role in neuronal development, synaptic transmission and regulation of many neuronal metabolic pathways (Missiaen et al., 2000). It was also reported that the perturbed cellular Ca^2+^ homeostasis correlates with amyloid plaques and neurofibrillary tangles in AD (Hölscher, 1998). Several studies further revealed that the neurotoxicity of Aβ was diminished if cells were incubated in Ca^2+^-free medium (Mattson et al., 1993) and Ca^2+^ from endoplasmic reticulum (ER) and mitochondria is involved into the pathogenesis of neuronal degeneration (Pereira et al., 2004; Takuma et al., 2005). Compounds that target neurotransmitters such as cholinergic receptors and 5-HT receptors may regulate the Ca^2+^ homeostasis, since Ca^2+^ signaling is initiated by neurotransmitters (Putney, 2003). Transient receptor potential (TRP) channels are plasma membrane cation channels consisting of six subfamilies: TRPA (ankyrin), TRPC (canonical), TRPM (melastatin), TRPML (mucolipin), TRPP (polycystin), and TRPV (vanilloid) (Moran, 2017). Aβ increases the production of ROS which further activates TRPC5, TRPM2, TRPM7, and TRPV1 and then triggers Ca^2+^ influx and induces NO production, finally leads to neurodegenerative and inflammatory processes (Yamamoto et al., 2007). In this case, TRPA1 was predicted as potential target for compounds TMGTY332, TMGTY293, and TMGTY291. TRPA1 is involved in the TRPA1-Ca^2+^-PP2B signaling cascade which contributes to Aβ-triggered inflammation and AD pathogenesis. Aβ can trigger TRPA1-dependent Ca^2+^ influx and then enhance the activity of protein phosphatase 2B (PP2B), which then activates NF-κB and nuclear factor of activated T cells (NFAT), leading to produce pro-inflammatory cytokines. The inhibition of TRPA1 channel can slow down AD progression (Lee et al., 2016). The predicted target genes of these 12 compounds including TPRA1, PTGS1, PTGS2, HTR2A, CHRM1, NOS2, and ALOX5 are all important protein-coding genes in these pathways related to AD. Hence the speculation can be made that this TCM formula may exert its therapeutic effect against AD by targeting those proteins to regulate Ca^2+^ and NO level and mollify neuroinflammation.

In order to further corroborate the prediction, a literature review was conducted to check if any of these 12 compounds is already experimentally validated to have therapeutic effect against AD. TMGTY404 (Dauricine) was reported to have neuroprotective effect, which could reduce energy depletion and oxidative stress, thus attenuate neuronal apoptotic cell death (Li and Gong, 2007). Dauricine was predicted to have interactions with six AD-related targets: MAO-A, AChE, dopamine receptor D1 (D_1_ receptor), 5-HT_1A_, MAO-B and beta-secretase 1 (BACE1). Targeting MAO-A and MAO-B could reduce the generation of ROS and targeting AChE, D_1_ receptor and 5-HT_1A_ together could improve cognitive performance. Thus Dauricine may exert neuroprotective effect through predicted AD-related targets. TMGTY115 (Apigenin) was also reported to have antioxidant and anti-inflammatory properties. Similarly, Apigenin could reduce ROS, protect from Aβ-induced toxicity and suppress inflammatory mediators such as NO and prostaglandin in rat and mouse cell experiments (Venigalla et al., 2015).

Above all indicated the formula TMGTY may treat AD through complex mechanisms, showing both neuroprotective and anti-neuroinflammatory effects.

### Overlapping Genes of AD and Hypertension Disease Modules

Nitric oxide plays a key role in the regulation of many physiological processes, such as vasodilation, inflammation, and neurodegeneration (Förstermann and Münzel, 2006; Brown, 2010). NO is generated by three NO synthase (NOS) isoforms: neuronal NOS (nNOS, encoded by NOS1), inducible NOS (iNOS, encoded by NOS2), and endothelial NOS (eNOS, encoded by NOS3). eNOS is constitutively expressed in the vascular endothelium where NO is continuously produced and involved in the regulation of vascular tone and blood pressure (Lundberg et al., 2015). In neurons, nNOS is activated by an influx of calcium to produce NO (Hall and Garthwaite, 2009). iNOS is highly expressed in inflammatory states and can produce high amounts of NO and other reactive nitrogen species such as peroxynitrite (Beckman et al., 1990). In diseased brain, iNOS is found mainly in microglia and astrocytes and may contribute to neuronal death and inflammatory neurodegeneration (Bal-Price and Brown, 2001; Brown and Bal-Price, 2003). So the overlapping genes may function in these enriched biological processes and involve in the regulation of blood pressure and NO levels in brain, thus contribute to the pathology of both AD and hypertension. Some other enriched biological processes such as positive regulation of neuron death and negative regulation of neuron apoptotic process suggested that these genes may also engage in the physiology of neuron cells.

These enriched biological processes further suggested that common disease genes should be investigated from a more systematic view. Among common disease genes, several genes were connected in protein–protein interaction network, which suggested that these genes may together exert certain biological effects. The analyses of these genes may also help to understand the concept ‘Syndrome’ in TCM. Hence, a sub-module was defined as a group of genes containing more than two connected gene nodes in disease modules, which was involved in specific biological processes. Genes enriched onto those above-mentioned biological processes were APOE, BCL2, CAT, ESR1, GPX1, GSK3B, IL1B, LEP, MTOR, NOS2, NOS3, SOD1, and SOD2. Eleven out of these 13 genes were from two sub-modules of common genes, i.e., sub-module 1 (AHR, APOE, ESR1, GSK3B, MTOR, NOS2, NOS3) and sub-module 2 (BCL2, CAT, GPX1, SOD1, SOD2). Drugs acting on common genes, especially genes in sub-modules, may show therapeutic effect on both AD and hypertension. Several genes from sub-modules were potential targets to the 12 representative compounds. ESR1 was predicted to interact with TMGTY405, TMGTY404, TMGTY115, and TMGTY387; BCL2 was the possible target of TMGTY408, TMGTY405, and TMGTY387; NOS2, NOS3 may interact with TMGTY067, and GSK3B with TMGTY115. Taking compound TMGTY067 (Angustidine) as an example, it was predicted to interact with two protein-coding genes NOS2 and NOS3 in the sub-module 1. NOS2 and NOS3 were involved in the regulation of blood pressure, removal of superoxide radicals and NO mediated signal transduction. Thus Angustidine may potentially regulate both the blood pressure and neuro-inflammation and worth further investigation on its pharmacological effects through biological experiments.

### The Synergistic Effect of TMGTY

**FIGURE 4:**
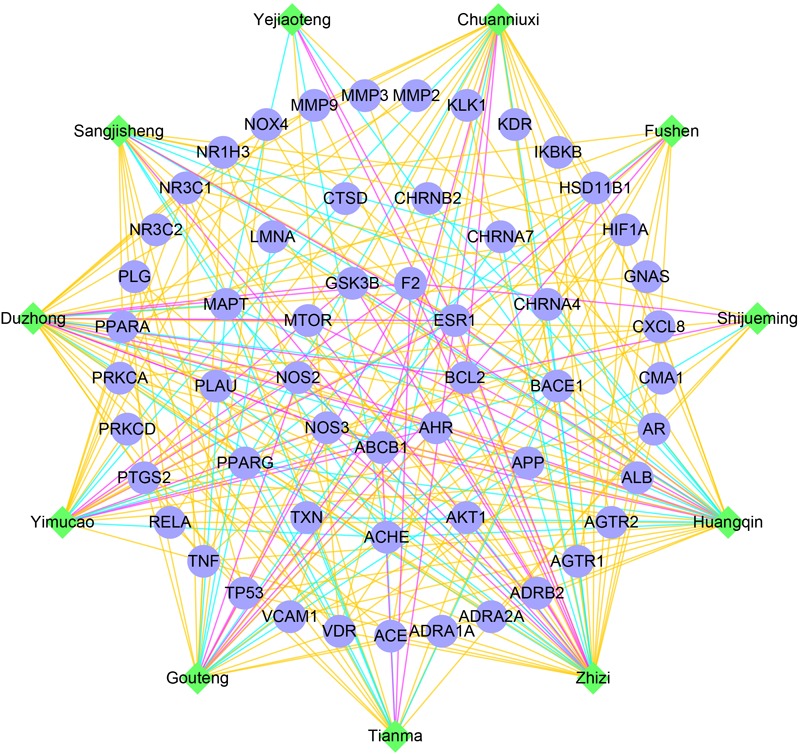
The network of TMGTY herbs (green nodes) and AD, hypertension related genes (blue nodes) in detected disease modules. Herbal interactions with AD genes were noted as cyan edges, hypertension genes as yellow edges and common genes were red edges.

Tian-Ma-Gou-Teng-Yin was prescribed for neurodegenerative diseases (Chik et al., 2013) such as AD (Liu et al., 2014; Lin et al., 2016) and Parkinson’s disease (PD) (Pan et al., 2015), and also the prevention of hypertension (Zhang et al., 2016). Upon previously detected disease modules, a network illustration of potential interactions between herbs and AD, hypertension related gene targets was constructed to speculate the synergistic effect of herb formula (**Figure 4**). According to node degrees, AD-related genes were usually potential targets of multiple herbs, such as ACHE (of 10 herbs), BACE1 (9), MAPT (9), and PPARG (8). Thus TMGTY may have a synergistic effect on neurotransmitter-involved pathways to alleviate symptoms of AD. Nine common disease genes in the disease relationship network were predicted to be targets of herb components. Six of them were in sub-module 1, i.e., ESR1 (10), NOS2 (5), NOS3 (5), AHR (5), GSK3B (4), and MTOR (3). All herbs had potential effect on both AD and hypertension disease modules. *Tianma* and *Gouteng* were reported to have anti-AD effects on *in vitro* or *in vivo* models (Su et al., 2014). The water extract of *Tianma* and *Gouteng* showed antioxidant and antiapoptotic effects on neuronal differentiated PC12 cells (Xian et al., 2016). *Yejiaoteng* was a commonly prescribed herb for the treatment of AD and sleeping disorder, and a *Yejiaoteng* decoction was also reported to have sedative-hypnotic effect in an animal model (Chen et al., 2015). Studies have shown that *Huangqin* had antioxidant and anti-neuroinflammatory effects in PC12 cells and mice models (Shang et al., 2006; Jeong et al., 2011). *Yimucao* extract was tested to have cerebral protective effect by reducing neurological impairment, oxidative damage and apoptosis in cerebral occluded rats (Loh et al., 2009). The extract of *Zhizi* also had antioxidant activity (Debnath et al., 2011). Five herbs, *Gouteng, Tianma, Yimucao, Zhizi*, and *Duzhong*, were predicted to interact with genes in sub-module 1. Many of these herbs were involved in the production of ROS, and had antioxidant effects. Genes in the sub-module 1 were directly enriched onto GO biological processes such as positive regulation of neuron death and NO mediated signal transduction. Thus acting on sub-module 1 to regulate NO-related oxidative state may be attributed to the formula’s common protective effects against both AD and hypertension.

A herb usually contains hundreds of compounds, and thus possesses a multi-target quality which results in multiple therapeutic effects. Subtly designed herb formulae consisting of several herbs may have synergistically enhanced therapeutic effects against certain symptoms. These symptoms may be a manifestation of functional gene groups. Thus a herb formula may possess complicated pharmacological activities.

## Conclusion

In this study, we proposed a computational systems pharmacology approach to investigate the molecular mechanisms of TCM formula TMGTY. We first collected the principal components of this formula and predicted targets for them. Then using hypergeometric distribution and Fisher’s exact test, those compounds were enriched onto AD through target proteins. The most representative compounds were selected and gene set enrichment analysis was conducted. Our approach revealed that formula TMGTY may have neuroprotective and anti-neuroinflammatory effects in treating AD. We further analyzed disease modules of AD and hypertension and found that some sub-modules of genes were shared between two diseases. Compounds (for example, Angustidine) targeting proteins in sub-modules (such as NOS2 and NOS3) may effect on both AD and hypertension.

Yet there are also limitations of our approach. To filter formula ingredients using predicted HIA and BBB properties may lead to an omission of some compounds. The incompleteness of herb ingredients, AD-related genes and PPI network would bring bias to the prediction and analysis. Furthermore our approach cannot discriminate whether a compound agonize or antagonize a target receptor, as well as the causal relationship between compounds and AD. For example, Isorhynchophylline and Gastrodin were not identified as top compounds since they were not predicted to have significant number of AD-related target genes. They had insufficient known target information and the prediction may be biased by above reasons. Thus the performance of our approach needs to be further improved, for example, by integrating drug-phenotype data. However, our approach is from a holistic perspective and easy to integrate new data to increase performance. At last but not least, our approach did not consider the quantity of each component in the formula, which is difficult currently and should be taken into account in future.

## Author Contributions

YT, GL, WL, and TW contributed to conception and design of the study. TW performed the experiments and wrote the manuscript. YT wrote sections of the manuscript. All authors contributed to manuscript revision, read and approved the submitted version.

## Conflict of Interest Statement

The authors declare that the research was conducted in the absence of any commercial or financial relationships that could be construed as a potential conflict of interest.

## Footnotes

1 http://lmmd.ecust.edu.cn/admetsar1/
