# Supplementary material for: A Computational Systems Pharmacology Approach to Investigate Molecular Mechanisms of Herbal Formula Tian-Ma-Gou-Teng-Yin for Treatment of Alzheimer’s Disease
Source: Front Pharmacol. 2018 Jun 26;9:668. doi: 10.3389/fphar.2018.00668 (PMC6028720; doi:10.3389/fphar.2018.00668)
Supplement: Supplementary file 2 [file Presentation_1.PDF]

Supplementary Material

A Computational Systems Pharmacology Approach to Investigate Molecular Mechanisms of Herbal Formula Tian-Ma-Gou-Teng-Yin for Treatment of Alzheimer's Disease

Tianduanyi Wang, Zengrui Wu, Lixia Sun, Weihua Li, Guixia Liu, Yun Tang*

Shanghai Key Laboratory of New Drug Design, School of Pharmacy, East China University of Science and Technology, Shanghai 200237, China

*Corresponding author, E-mail: ytang234@ecust.edu.cn

1. Supplementary Data

Case study 2. Analysis of herbal formula Kai-Xin-San

Kai-Xin-San (KXS) is a famous traditional Chinese herbal formula used for the treatment of neurasthenia, neurosis, depression and Alzheimer's disease(Mu *et al.*, 2011; Zhou *et al.*, 2012; Wang *et al.*, 2015; Chu *et al.*, 2016). KXS consists of four herbs: *Panax ginseng (Renshen), Wolfiporia cocos (Fuling), Polygala tenuifolia (Yuanzhi) and Acorus tatarinowii (Shichangpu).*

The systems network pharmacology approach was applied to formula KXS to analyze its pharmacological effects.

A total of 503 herbal components were collected for four herbs. After BBB and HIA screening, 388 compounds were retained (Supplementary Table S9). 38 out of 388 compounds had 124 known targets, which formed 201 known compound-target interactions. Then 369 new targets were predicted and 7760 potential interactions were introduced. All known and predicted targets of KXS were listed in Supplementary Table S10. 28 representative compounds were identified through Fisher's exact test (adjusted p-value < 0.05, Supplementary Table S11). Known and predicted targets of these 28 compounds were enriched onto KEGG pathway and GO biological process (Supplementary Table S12 and S13). Compound names and structures were shown in Supplementary Figure S6.

Among enriched pathways, nitrogen metabolism (adjusted p-value = 2.17×10^{-5}), neuroprotective ligand-receptor interaction (adjusted p-value = 1.33×10^{-5}), serotonergic synapse (adjusted p-value = 3.69×10^{-5}), dopaminergic synapse (adjusted p-value = 0.012), arachidonic acid metabolism (adjusted p-value = 1.99×10^{-5}), linoleic acid metabolism (adjusted p-value = 0.013), and tryptophan metabolism (adjusted p-value = 0.037) may be involved in AD pathology. GO biological process enrichment analysis indicated that KXS may involve in oxidation-reduction process (adjusted p-value = 1.80×10^{-13}), steroid metabolic process (adjusted p-value = 5.38×10^{-6}), monoterpenoid metabolic process (adjusted p-value = 6.36×10^{-6}), memory (adjusted p-value = 4.28×10^{-5}), heterocycle metabolic process (adjusted p-value = 7.44×10^{-4}), lipoxygenase pathway (adjusted p-value = 0.0068) and synaptic transmission, dopaminergic (adjusted p-value = 0.0068). These enrichment analyses suggested that KXS may exert neuroprotective effect by regulating metabolism networks, reversing oxidative damage in brain, as well as targeting neurotransmitter pathways.

Chu et al. discovered that KXS could alleviate cognitive deficits in AD model rats and more nerve cells survived than that in the control group. They also found that KXS could restore several disturbed metabolic pathways, such as linoleic acid metabolism, arachidonic acid metabolism, and sphingolipid metabolism, to exert protective effects in AD progression. Linoleic acid plays an important role in protecting brain from

neurodegenerative processes(Beydoun *et al.*, 2007). Oleamide is an amide lipid found to interact with neurotransmitter systems and could increase choline acetyltransferase activity in vitro(Fedorova *et al.*, 2001; Heo *et al.*, 2003). Thus they concluded that KXS could regulate metabolism network by affecting certain metabolites to show anti-AD effects(Chu *et al.*, 2016).

Qiong et al. found that in rat models KXS could reduce the level of 3-nitro tyrosine (3-NT), which is a marker of protein oxidation injury. KXS could also increase the activity of choline acetyltransferase. Thus their results indicated antioxidant effects of KXS(Qiong *et al.*, 2016). Lu et al. reported that KXS could restore the deficiency in cholinergic and glutamatergic neurotransmission and enhance the expression of downstream signal transduction molecules in hippocampus, thus ameliorate Aβ-induced cognitive impairment(Lu *et al.*, 2017). Zhu et al. discovered that KXS could induce synaptic protein expression in hippocampus neuron in rats, and they also found that KXS could enhance the expression levels of neurofilaments, decrease the expression of neurotransmitter degradation enzymes and induce neuronal differentiation in PC12 cells(Zhu *et al.*, 2016a; Zhu *et al.*, 2016b).

In 28 representative compounds, Apigenin, Paeonol were reported to be important anti-AD compounds(Su *et al.*, 2014). Eudesmin could up-regulate the expression of $GABA_A$ and Bcl-2, and it has significant anticonvulsant and sedative effects(Liu *et al.*, 2015). 2'-O-Methylisoliquiritigenin was reported to have antioxidant activity and it was also active against human neuroblastoma cells (IC_{50} = 32.5μM)(Batovska & Todorova, 2010). Deng et al. also reported that 2'-O-Methylisoliquiritigenin may have a sedative−hypnotic effect by affecting neurotransmitter levels in mice(Klein & Lindell, 2014). Marmesin was reported to have AChE inhibitory effects(Cabral *et al.*, 2012; Tumiatti *et al.*, 2008). Bergapten was discovered to have anti-inflammatory effects by suppressing the ROS and NO generation(Yang *et al.*, 2018), and it may also have inhibitory effect against BChE(Erdogan Orhan, 2012). Myrcene was reported to have significant anti-inflammatory and anti-catabolic activities(Rufino *et al.*, 2015). Eugenol was also reported to have anti-inflammatory and anti-oxidative activities. Furthermore, Eugenol could inhibit Aβ-induced excessive influx of calcium ion into neurons, and possess antidepressant-like activity(Irie, 2006).

Above analyses further proved that our systems network pharmacology approach would be useful in analyzing polypharmacological effects of TCM formulae and identifying key herbal constituents.

However, gensenosides were not identified, which may due to their huge molecular weight and TPSA values. Most gensenosides had molecular weights larger than 600 and TPSA values larger than 200, and some compounds' molecular weights even larger than 1000, which lay beyond the chemical space of our Global network model. In our Global model, only 199 out of 3880 compounds had molecular weights larger than 600 and only 10 compounds larger than 1000. As for TPSA values, only 181

compounds were larger than 200 and only 47 compounds larger than 47. Only 96 compounds had molecular weights larger than 600 while TPSA values larger than 200. Thus the performance of our prediction model was diminished when applying it to compounds having very large molecular weights.

References:

Batovska, D.I., and Todorova, I.T. (2010). Trends in Utilization of the Pharmacological Potential of Chalcones. *Current Clinical Pharmacology* 5(1)**,** 1-29. doi: 10.2174/157488410790410579.

Beydoun, M.A., Kaufman, J.S., Satia, J.A., Rosamond, W., and Folsom, A.R. (2007). Plasma n-3 fatty acids and the risk of cognitive decline in older adults: The atherosclerosis risk in communities study. *American Journal of Clinical Nutrition* 85(4)**,** 1103-1111.

Cabral, R.S., Sartori, M.C., Cordeiro, I., Queiroga, C.L., Eberlin, M.N., Lago, J.H.G., et al. (2012). Anticholinesterase activity evaluation of alkaloids and coumarin from stems of Conchocarpus fontanesianus. *Revista Brasileira de Farmacognosia* 22**,** 374-380.

Chu, H., Zhang, A., Han, Y., Lu, S., Kong, L., Han, J., et al. (2016). Metabolomics approach to explore the effects of Kai-Xin-San on Alzheimer's disease using UPLC/ESI-Q-TOF mass spectrometry. *Journal of Chromatography B* 1015-1016**,** 50-61. doi: https://doi.org/10.1016/j.jchromb.2016.02.007.

Erdogan Orhan, I. (2012). Current Concepts on Selected Plant Secondary Metabolites With Promising Inhibitory Effects Against Enzymes Linked to Alzheimer’s Disease. *Current Medicinal Chemistry* 19(14)**,** 2252-2261. doi: 10.2174/092986712800229032.

Fedorova, I., Hashimoto, A., Fecik, R.A., Hedrick, M.P., Hanuš, L.O., Boger, D.L., et al. (2001). Behavioral evidence for the interaction of oleamide with multiple neurotransmitter systems. *Journal of Pharmacology and Experimental Therapeutics* 299(1)**,** 332-342.

Heo, H.-J., Park, Y.-J., Suh, Y.-M., Choi, S.-J., Kim, M.-J., Cho, H.-Y., et al. (2003). Effects of Oleamide on Choline Acetyltransferase and Cognitive Activities. *Bioscience, Biotechnology, and Biochemistry* 67(6)**,** 1284-1291. doi: 10.1271/bbb.67.1284.

Irie, Y. (2006). Effects of Eugenol on the Central Nervous System: Its Possible Application to Treatment of Alzheimer's Disease, Depression, and Parkinson's Disease. Current Bioactive Compounds 2(1), 57-66.

Deng, L. , Shi, A. and Wang, Q. (2018). Sedative‐Hypnotic and Anxiolytic Effects and the Mechanism of Action of Aqueous Extracts of Peanut Stems and Leaves in Mice. *Journal of the Science of Food and Agriculture*. Accepted Author Manuscript. doi: 10.1002/jsfa.9020

Liu, H., Song, Z., Liao, D.G., Zhang, T.Y., Liu, F., Zhuang, K., et al. (2015). Anticonvulsant and Sedative Effects of Eudesmin isolated from Acorus tatarinowii on mice and rats. *Phytotherapy Research* 29(7)**,** 996-1003. doi: 10.1002/ptr.5337.

Lu, C., Shi, Z., Sun, X., Pan, R., Chen, S., Li, Y., et al. (2017). Kai Xin San aqueous extract improves Aβ1-40-induced cognitive deficits on adaptive behavior learning by enhancing memory-related molecules expression in the hippocampus. *Journal of Ethnopharmacology* 201**,** 73-81. doi: https://doi.org/10.1016/j.jep.2016.10.002.

Mu, L.-H., Huang, Z.-X., Liu, P., Hu, Y., and Gao, Y. (2011). Acute and subchronic oral toxicity

assessment of the herbal formula Kai-Xin-San. *Journal of Ethnopharmacology* 138(2)**,** 351-357. doi: https://doi.org/10.1016/j.jep.2011.08.033.

Qiong, W., Yong-liang, Z., Ying-hui, L., Shan-guang, C., Jiang-hui, G., Yi-Xi, C., et al. (2016). The memory enhancement effect of Kai Xin San on cognitive deficit induced by simulated weightlessness in rats. *Journal of Ethnopharmacology* 187**,** 9-16. doi: https://doi.org/10.1016/j.jep.2016.03.070.

Rufino, A.T., Ribeiro, M., Sousa, C., Judas, F., Salgueiro, L., Cavaleiro, C., et al. (2015). Evaluation of the anti-inflammatory, anti-catabolic and pro-anabolic effects of E-caryophyllene, myrcene and limonene in a cell model of osteoarthritis. *European Journal of Pharmacology* 750**,** 141-150. doi: https://doi.org/10.1016/j.ejphar.2015.01.018.

Su, Y., Wang, Q., Wang, C., Chan, K., Sun, Y., and Kuang, H. (2014). The treatment of Alzheimer's disease using Chinese Medicinal Plants: From disease models to potential clinical applications. *Journal of Ethnopharmacology* 152(3)**,** 403-423. doi: https://doi.org/10.1016/j.jep.2013.12.053.

Tumiatti, V., Bolognesi, M.L., Minarini, A., Rosini, M., Milelli, A., Matera, R., et al. (2008). Progress in acetylcholinesterase inhibitors for Alzheimer's disease: an update. *Expert Opinion on Therapeutic Patents* 18(4)**,** 387-401. doi: 10.1517/13543776.18.4.387.

Wang, X., Han, Y., Zhang, A., and Sun, H. (2015). "Chapter 22 - Metabolic Profiling Provides a System for the Understanding of Alzheimer’s Disease in Rats Post-Treatment With Kaixin San," in *Chinmedomics*. (Boston: Academic Press), 347-362.

Yang, Y., Zheng, K., Mei, W., Wang, Y., Yu, C., Yu, B., et al. (2018). Anti-inflammatory and proresolution activities of bergapten isolated from the roots of Ficus hirta in an in vivo zebrafish model. *Biochemical and Biophysical Research Communications* 496(2)**,** 763-769. doi: https://doi.org/10.1016/j.bbrc.2018.01.071.

Zhou, X.-J., Liu, M., Yan, J.-J., Cao, Y., and Liu, P. (2012). Antidepressant-like effect of the extracted of Kai Xin San, a traditional Chinese herbal prescription, is explained by modulation of the central monoaminergic neurotransmitter system in mouse. *Journal of Ethnopharmacology* 139(2)**,** 422-428. doi: https://doi.org/10.1016/j.jep.2011.11.027.

Zhu, Y., Duan, X., Cheng, X., Cheng, X., Li, X., Zhang, L., et al. (2016a). Kai-Xin-San, a standardized traditional Chinese medicine formula, up-regulates the expressions of synaptic proteins on hippocampus of chronic mild stress induced depressive rats and primary cultured rat hippocampal neuron. *Journal of Ethnopharmacology* 193**,** 423-432. doi: https://doi.org/10.1016/j.jep.2016.09.037.

Zhu, Y., Duan, X., Huang, F., Cheng, X., Zhang, L., Liu, P., et al. (2016b). Kai-Xin-San, a traditional Chinese medicine formula, induces neuronal differentiation of cultured PC12 cells: Modulating neurotransmitter regulation enzymes and potentiating NGF inducing neurite outgrowth. *Journal of Ethnopharmacology* 193**,** 272-282. doi: https://doi.org/10.1016/j.jep.2016.08.013.

2. Supplementary Figures

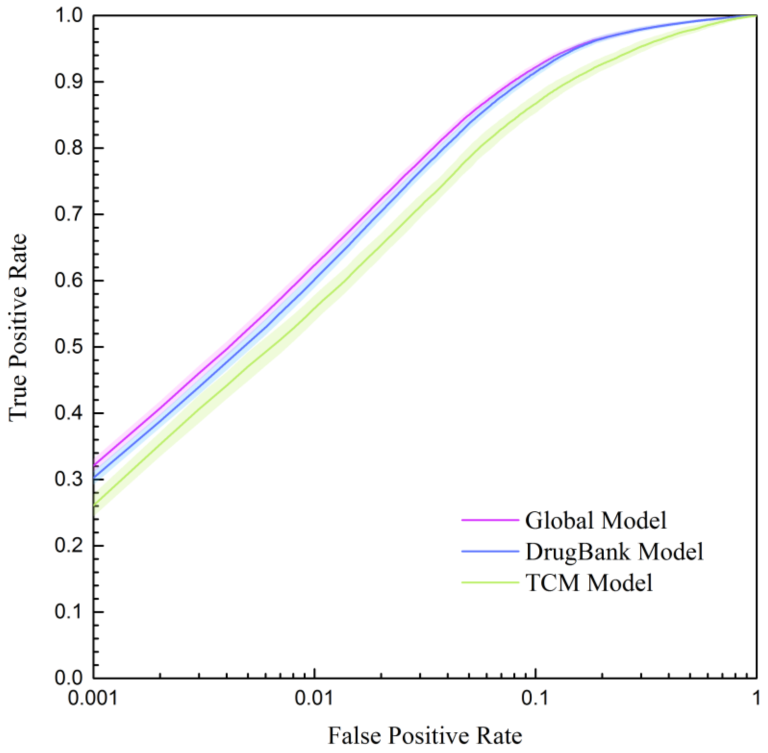

Supplementary Figure S1. The ROC curves of three network models.

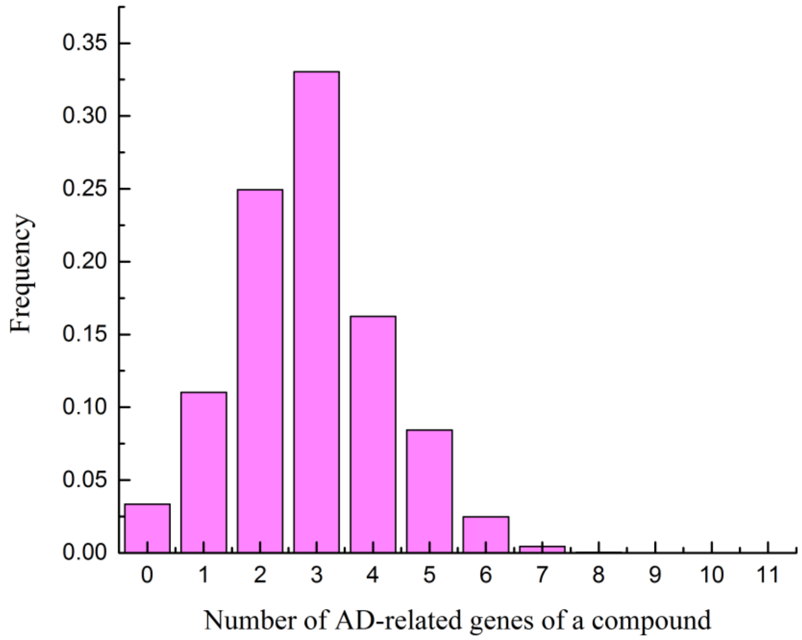

Supplementary Figure S2. The distribution of number of AD-related genes in the background compound set.

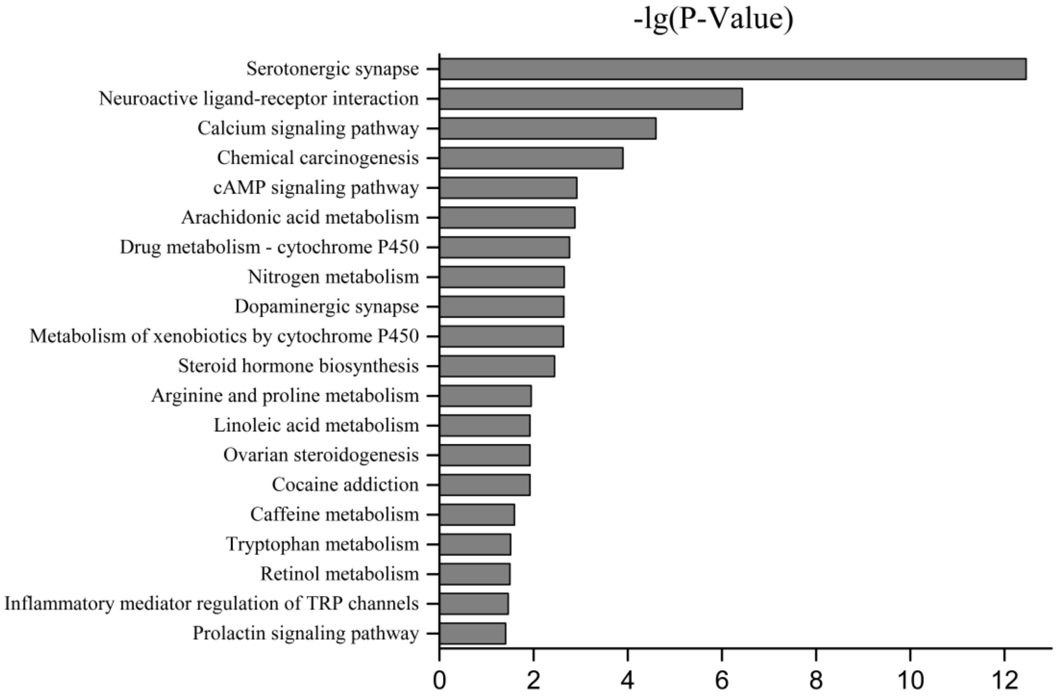

Supplementary Figure S3. Gene set enrichment analysis results of KEGG pathways for overall targets of 12 compounds (p-value < 0.05).

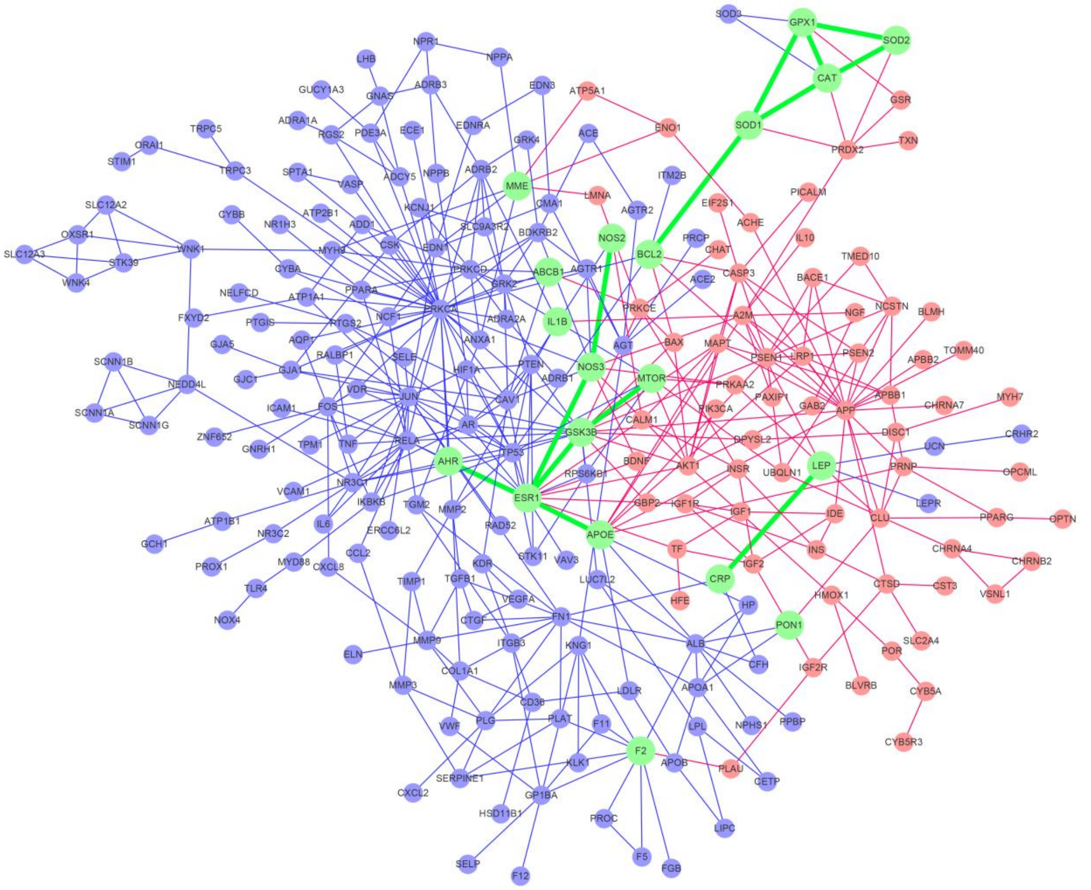

Supplementary Figure S4. The disease modules of AD (red nodes) and hypertension (blue nodes) and their overlapping genes (green nodes).

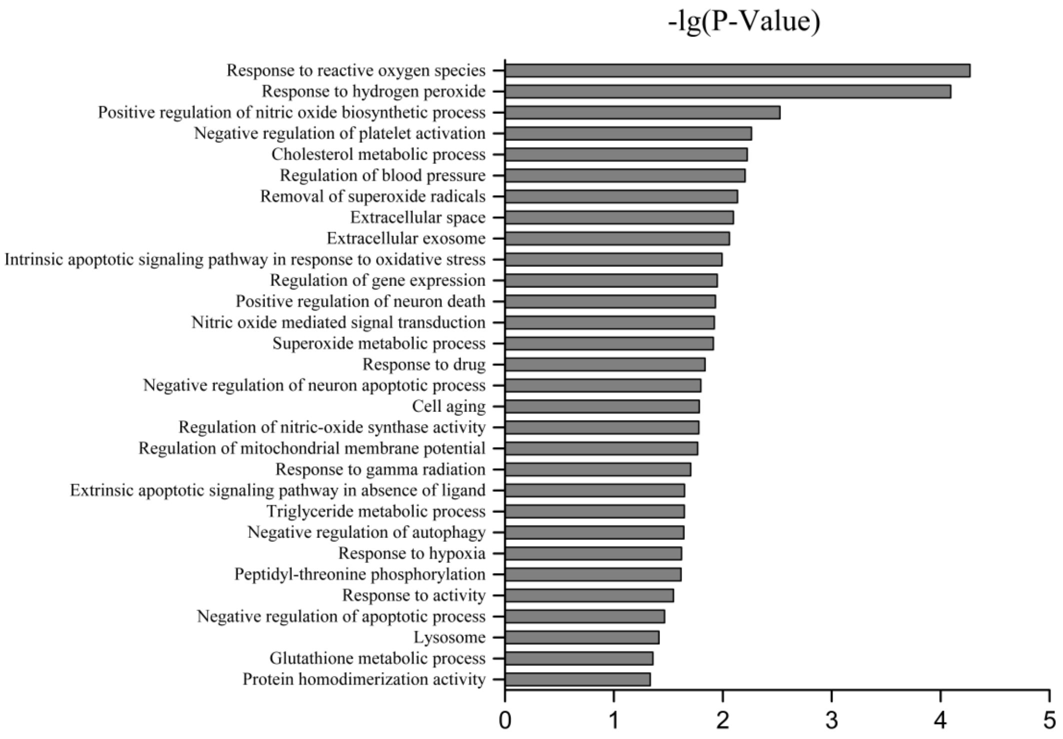

Supplementary Figure S5. GO biological process enrichment results for 19 overlapping genes of AD and hypertension disease modules (p-value < 0.05).

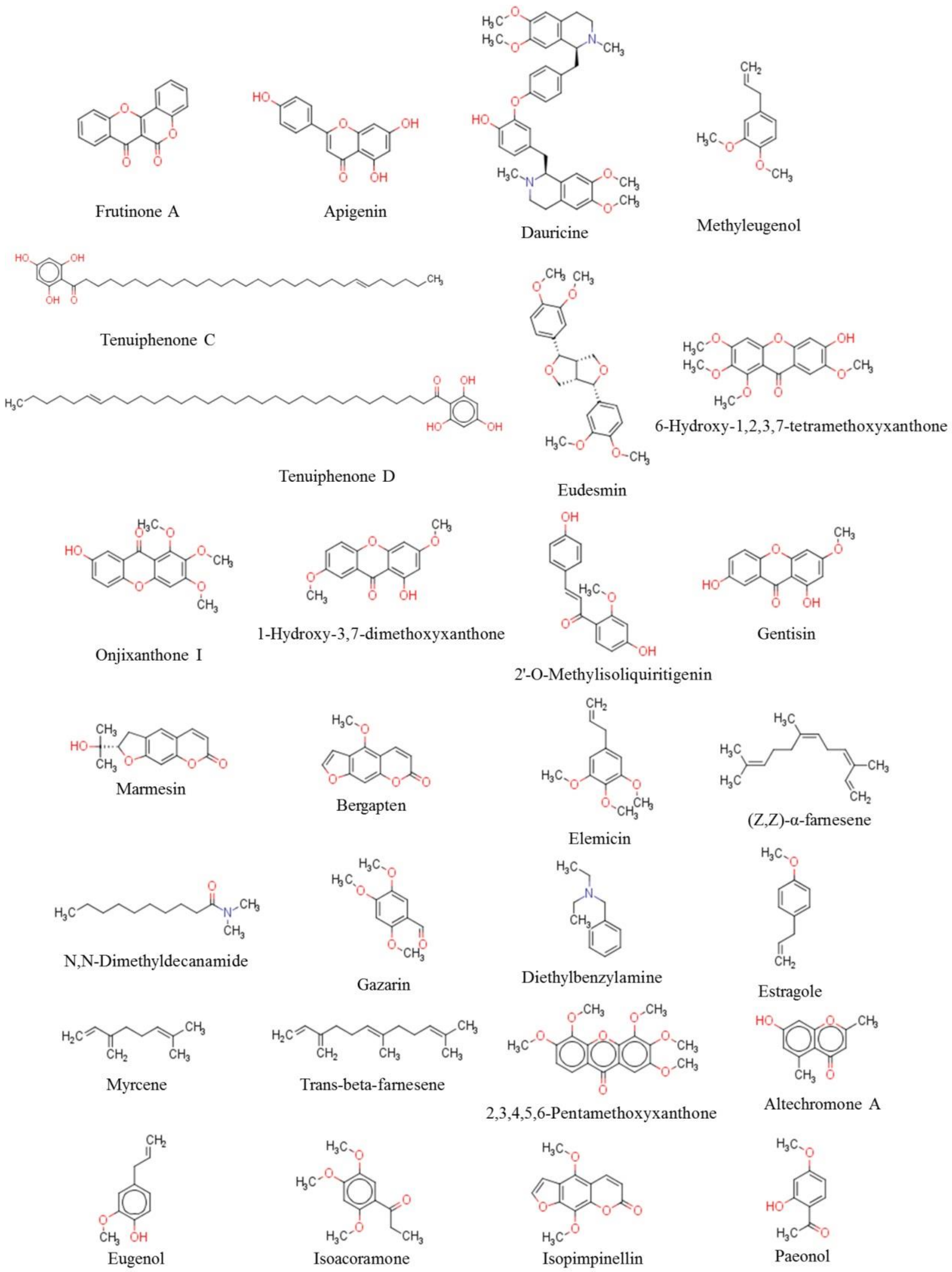

Supplementary Figure S6. Names and structures of 28 representative compounds of KXS.
